# Immunity in Gilles de la Tourette-Syndrome: Results From a Cerebrospinal Fluid Study

**DOI:** 10.3389/fneur.2019.00732

**Published:** 2019-07-04

**Authors:** Charlotte Baumgaertel, Thomas Skripuletz, Jessica Kronenberg, Martin Stangel, Philipp Schwenkenbecher, Christopher Sinke, Kirsten R. Müller-Vahl, Kurt-Wolfram Sühs

**Affiliations:** ^1^Department of Psychiatry, Social Psychiatry and Psychotherapy, Hannover Medical School, Hanover, Germany; ^2^Department of Neurology, Hannover Medical School, Hanover, Germany; ^3^Center for Systems Neuroscience, University of Veterinary Medicine Hannover, Hanover, Germany

**Keywords:** Tourette-syndrome, autoimmunity, cerebrospinal fluid, oligoclonal bands, antibodies, tics, immunology

## Abstract

**Background:** Several lines of evidence support the hypothesis of an autoimmune origin of Gilles de la Tourette-Syndrome (GTS). Accordingly, in a recent study we detected positive oligoclonal bands (OCB) in cerebrospinal fluid (CSF) in >30% of adult patients indicating an intrathecal antibody synthesis. However, until today no corresponding antibodies could be identified. The aims of this study were to replicate our findings of positive OCB in an independent sample and to detect CSF autoantibodies.

**Methods:** In this prospective study, 20 adult patients with GTS (male: female = 18:2, median age 36.1 years ± 14.34 SD) were included. All patients were thoroughly clinically characterized. Magnetic Resonance Imaging (MRI) and CSF standard measurements were performed. Isoelectric focusing on polyacrylamide gels with silver staining was used to detect OCB. To examine specific and unspecified autoantibodies, we used transfected Human Embryonic Kidney (HEK) cells expressing different surface antigens (NMDA-, CASPR2-, LGI1-, AMPA-, or GABAB1/B), indirect immunofluorescence on different brain tissue sections, and enzyme-linked visualization. Additionally, we differentiated Glioma stem cells SY5Y (human neuroblastoma) using retinoic acid and astrocytes (rat).

**Results:** CSF analyses showed positive OCB (type 2) in 4/20 patients (20%). Using transfected HEK cells we did not find specific surface-autoantibodies. Immunohistochemistry on tissue-sections, SY5Y Glioma stem-cells, and astrocytes showed no specific binding patterns either.

**Conclusions:** Our results corroborate previous findings and demonstrate positive OCB in a substantial number of patients with GTS (prevalence in healthy controls: 5%). Although this is the largest study investigating CSF autoantibodies in GTS using several techniques, we failed to detect any specific or unspecified autoantibodies.

## Introduction

Gilles de la Tourette-Syndrome (GTS) is a neuropsychiatric disorder characterized by childhood onset motor and vocal tics (DSM-5) that fluctuate spontaneously over time (1). It is thought that GTS is caused by alterations in cortico-striato-thalamo-cortical circuits. Several lines of evidence suggest that both genetic and non-genetic influences contribute to the etiology of GTS (2, 3). It has been demonstrated that multiple common genetic variants of small effect play a role, but in recent genome-wide association studies (GWASs) no single-nucleotide polymorphisms (SNPs) met criteria for genome-wide significance (4, 5). While in a first genome-wide *epigenetic* analysis, no methylation site reached significance (6), altered methylation levels of different dopaminergic genes (dopamine D2 receptor, DRD2, dopamine transporter, DAT) could be detected, when measuring peripheral DNA methylation (7). Finally, several interacting environmental factors seem to be involved in the pathogenesis of GTS such as psychosocial stress (8), perinatal risk factors (9), and immunological changes (10). Accordingly, several different abnormalities in the peripheral immune system have been described including increased serum levels of Tumor Necrosis Factor-Alpha (TNF-α), Interleukin 12 (IL-12) (11) and several other interleukins (IL) such as IL-6, IL-8, IL-1β, and IL-17 as well as interferon-gamma induced protein 10 k (IP-10), an indicator for activation of cellular immunity (12). Furthermore, increased levels of antinuclear antibodies (ANA) (13), C-reactive protein (CRP), and neopterin, increased numbers of monocytes (14), increased concentrations of CD4-, CD95-, CD8-, CD69-, B-, and T-cells, and an overexpression of natural killer (NK)-cells (15) in patients' sera suggest increased inflammatory activity in patients with GTS. Accordingly, in an animal model for GTS, striatal dysfunction could be provoked by intrastriatal microinfusion of sera from patients with GTS (16) suggesting abnormalities in the immune response in the central nervous system. However, there is only one study examining inflammatory changes in cerebrospinal fluid (CSF) ((17), see below) whereas most other studies examined patients sera, and therefore assessed changes in the peripheral immune system.

In line with these findings, the PANDAS (= Pediatric Autoimmune Neuropsychiatric Disorders Associated with Streptococcal infections) concept has been suggested, based on the hypothesis that diseases associated with tics and/or obsessive compulsive disorder (OCD) might be caused by group A streptococcal (GAS) infections (18). The fact that many patients with a clinically similar syndrome to PANDAS have no evidence of streptococcal infection, resulted in the generation of the term PANS (= Pediatric Acute-onset Neuropsychiatric Syndrome) describing a syndrome with abrupt onset of obsessive-compulsive symptoms, anxiety, and sensory symptoms in previously healthy children (19). However, results from the recently completed European-wide EMTICS study (20) failed to demonstrate evidence for a causal role of streptococcal and non-streptococcal bacteria in the onset or exacerbation of tics, but confirmed recent data for an abnormal immune responsiveness in patients with GTS with lower levels of pro-inflammatory cytokines IL-6 and TNF-α and soluble TNF-receptor as well as higher immunoglobulin levels soluble monocytes activation marker CD14 (21).

In order to further explore immunological changes in GTS, in a recent study, we analyzed autoantibodies in sera of 51 patients, but failed to detect any abnormalities for N-methyl-D-aspartic acid- (NMDA), contactin-associated protein related 2- (CASPR2), Leucin-rich glioma inactivated protein (LGI1), or gamma-aminobutyric acid (GABAB1/B2) (22). In contrast, Dale et al. (23) reported elevated levels of antibodies against dopamine-2 receptors in 4/44 of patients with GTS.

Unfortunately, the vast majority of studies investigating antibodies in GTS is limited by the fact that blood sera have been used, but not cerebrospinal fluid (CSF). This is of paramount importance, since in neuropsychiatric disorders results from CSF are more meaningful, not only because the blood-brain barrier segregates blood from CSF limiting antibodies and other determinants to pass, but also the fact that CSF contains less proteins than blood, which may influence the results. Even though in a recent case-control study in five children with GTS (age range, 6–12 years) no CSF specific oligoclonal bands (OCB) could be detected (17), in a recently performed larger study in a mixed population (median age 29 ± 12 SD, range, 9–51 years), we found positive CSF OCB in 8/21 (38%) of patients (24). This finding further corroborates an autoimmune hypothesis in GTS, since OCB are IgG antibodies produced by plasma cells that are found in only 5% of healthy people (25) and therefore document—in nearly all cases—a pathological antibody synthesis within the central nervous system.

The aim of this study was to perform extensive CSF analyses in a group of adult patients with GTS. We expected to replicate recent findings of positive OCB (24) and to detect CSF autoantibodies particularly in those patients with positive OCB.

## Methods

### Patients

In this prospective study, we included 20 adult patients (male: female = 18:2) with GTS according to DSM-5. In all cases, the diagnosis was confirmed by one of the authors (KMV). Patients were recruited from our Tourette outpatient clinic at the Hannover Medical School and internet calls between 10/2015 and 3/2016. All examinations were conducted in accordance with the declaration of Helsinki and after approval of the Local Ethics Committee (no. 6987) of the Hannover Medical School. All patients gave written informed consent before entering the study. The inclusion criteria were: (i) age >18 years, (ii) confirmed diagnosis of GTS, (iii) no other severe psychiatric or neurological diseases such as schizophrenia, alcoholism, epilepsy, and mental retardation as well as no (autoimmune) diseases that are known to cause CSF changes including OCB. Additionally, all patients were screened for contraindications of a lumbar puncture such as disturbances of blood coagulation (including measurement of International Normalized Ratio (INR), Partial Thromboplastin Time (PTT), and thrombocyte count), increased intracranial pressure, and spinal tumors.

### Magnetic Resonance Imaging (MRI)

Before lumbar puncture, in all patients cerebral Magnetic Resonance Imaging (MRI) was performed to exclude (i) brain pathologies associated with increased intracranial pressure, and (ii) immunological diseases known to cause positive OCB such as multiple sclerosis (MS). All images were acquired on a 3T Siemens Skyra MRI scanner equipped with 32 channel head coils. For structural images, T1 weighted images were acquired by means of a MPRAGE sequence (slices = 192, FoV = 256 mm, voxel size = 1 × 1 × 1 mm, TR = 2.5 s, TE = 4.37 ms, flip angle = 7°, distance factor = 50%).

### Clinical Assessments

All patients were carefully clinically assessed using the following standardized assessments: (i) Yale Global Tic Severity Scale—otal Tic Score (YGTSS-TTS) (26) to measure tic severity, (ii) Premonitory Urge for Tics Scale (PUTS) (27) to assess tic-related urges, and (iii) Gilles de la Tourette-Syndrome Quality of Life Scale (GTS-QOL) (28) to measure patients health-related quality of life. To assess common comorbidities, we used (i) Beck Depression Inventory-II (BDI-II) (29), (ii) Conner's Adult Attention deficit/hyperactivity disorder (ADHD) Rating Scale (CAARS) (30), (iii) DSM-IV symptom list for ADHD (31), (iv) Wender Utah Rating Scale short version (WURS-K) (32), (v) Beck Anxiety Inventory (BAI) (33), (vi) Yale Brown Obsessive Compulsive Scale (Y-BOCS) (34), and (vii) Brief Symptom Inventory (BSI) for psychological distress and psychiatric disorders (35).

### CSF Analysis

After lumbar puncture, CSF samples were immediately frozen at −80°C for up to 4 months prior to analyses.

#### Routine Parameters

CSF was analyzed by standard methods assessing routine CSF parameters including (i) CSF cell count using a Fuchs-Rosenthal counting chamber, (ii) manual assessment of cytology and (iii) blood-CSF barrier function by CSF-serum albumin quotients [QAlb (36, 37)]. The age-adjusted upper reference limit of QAlb was calculated using the following formula: QAlb = 4 + (age in years/15) [formula of Reiber-Felgenhauer (38)], and (iv) OCB by isoelectric focusing on macro polyacrylamide gels with consecutive silver staining simultaneously in CSF and blood sera (39).

#### Detection of Specific CSF Autoantibodies

For the assessment of well-known specific CSF autoantibodies, we used transfected Human Embryonic Kidney Cells (HEK293, Autoimmune-Encephalitis-Mosaik 1 FA 112D 1003-1, Euroimmun, Lübeck, Germany), which express either NMDA-, CASPR2-, LGI1-, AMPA- or GABAB1/B2. The HEK-cells were incubated with 30 μl undiluted CSF and washed afterwards to detect antibodies in CSF. Bound autoantibodies in CSF were labeled with secondary fluorescein-conjugated goat anti-human antibodies (Mosaik 1 FA 112D 1003-1, Euroimmun, Lübeck, Germany). As a positive control we used the NMDAR antibody provided in the kit (Mosaik 1 FA 112D 1003-1, Euroimmun, Lübeck, Germany).

#### Detection of Unspecified CSF Autoantibodies

To detect antibodies targeting unknown antigens, we used indirect immunofluorescence and incubated (30 min) tissue sections from monkey cerebellum (all tissues: Glutamate-Receptor-Mosaik 3 FA 111m 1003-3, Euroimmun, Lübeck, Germany), hippocampus (rat), basal ganglia (rat), and Eu90-cells (as negative control) with undiluted patient CSF. In order to visualize bound antibodies, we used fluorescein isothiocyanate (FITC) marked anti-human IgG (Mosaik 3 FA 111m 1003-3, Euroimmun, Lübeck, Germany) provided by the kit as secondary antibody. To control for immunofluorescence staining artifacts, we used enzyme-linked visualization by incubating tissue sections from cerebellum (monkey) with 1:1 diluted patient CSF and anti-human IgG antibodies, adding 3 3′-diaminobenzidine (DAB) as dye (Vector Laboratories, Burlingame, USA). 1:100 diluted Anti-Yo and anti-Hu positive sera (of patients with immunoblot-confirmed antibodies) were used as positive controls. Antinuclear antibodies were described if a typical nuclear binding to all cell nuclei was present in tissue sections.

#### Glioma Stem Cell Line

To measure IgG binding to cell surface neuronal antigens, we differentiated a human Glioma cell line (neuroblastoma, SY5Y). Therefore, SY5Y cells were incubated for 3 days in nutritional medium with 10 μl retinoic acid (DMEM/F-12, ThermoFisher Scientific). After plating on poly-l-lysine-coated wells (20.000 cells/well), we incubated the cells with 200 μl undiluted patient CSF. Antibody binding was visualized by fluorescing goat-anti human IgG (Alexa Fluor 488, Invitrogen, 1:500) as secondary antibody. Cells were double labeled using rabbit-anti Microtubule-Associated Protein 2 (map-2, Millipore, 1:500) as second primary antibody and fluorescing goat-anti rabbit (Alexa Fluor 555, Invitrogen, 1:500) as second secondary antibody. Cells incubated with Hu- and Yo-receptor positive sera (patient sera with immunoblot-confirmed antibodies), diluted 1:10 using phosphate buffered saline and triton 0.1%, served as positive controls.

#### Glia Cells: Astrocytes

Since astrocytes closely interact with neurons and regulate synaptic transmission and plasticity, we additionally assessed antibody binding on this cell type. On that account, we prepared astrocytes from newborn rat brains. Preparations were done in accordance with the international guidelines for the use of laboratory animals. Therefore, 50,000 astrocytes/well were plated and incubated with nutritional medium (DMEM + 1% Pen/Strep + 10% FCS) for 3 days. Assessed by Glial Fibrillary Acid Protein, the purity of astrocyte cultures was over 95%. For detection of antibody binding, the astrocytes were incubated with undiluted patient CSF and fluorescing goat anti-human IgG (Alexa Fluor 488, Invitrogen, 1:500) as secondary antibody. Anti-Glial Fibrillary Acid Protein antibodies (GFAP, Millipore, 1:200) as a second primary antibody and fluorescing goat anti-rabbit (Invitrogen, Alexa Fluor 555, 1:500) as a second secondary antibody were used to label astrocytes. As positive control we used aquaporin 4 (AP4) positive serum (patient sera with confirmed AP4-antibodies, 1:200). Additionally, CSF of all patients were examined for AP4- and MOG-antibodies using a commercially available AP4-antibody kit (Aquaporin4 FA1128-1005-1, Euroimmun, Lübeck, Germany).

### Blood Sera

Referring to the PANDAS hypothesis of streptococcal infections in GTS, in addition, we measured serum anti-streptolysin titers. Based on recent findings of positive dopamine-2 receptor antibodies (23), we incubated SY5Y cells with 1:10 diluted patient's sera and compared binding patterns with a D2-positive control (primary antibody mouse anti-human D2 IgG, 1:500; secondary antibody goat anti-mouse IgG, 488, 1:500).

### Statistical Analyzes

All statistics were calculated using SPSS. For statistical analysis a *p*-value < 0.05 was considered significant. All statistical analyses were performed using two-tailed testing. As all dependent variables were normally distributed (tested using Kolmogorow-Smirnow test), parametric tests were used throughout. Due to the small sample sizes, we assumed variance homogeneity for all tests.

## Results

### Clinical Assessments

We included 20 patients with GTS (median age 36.1 ± 14.34 SD, range, 19–64 years) with a mean age at tic onset of 7.7 years (±2.8 SD, range, 3–13 years). Mean tic severity according to YGTSS-TTS was 23.2 (±9.1 SD, range, 10–39). All other clinical details are summarized in Table 1.

**Table 1:** Clinical characteristics of patients.

**Type of OCB**	**Age- range**	**No**.	**Albumin quotient Q(Alb) CSF/Ser**	**Streptolysine titer** (IU/ml)^**^	**ANA**	**Medication**	**Age at onset of tics**	**YGTSS-TTS (range 0–50)**	**Y-BOCS** **(range 0–40)**	**PUTS (range 10–40)**	**GTS-QoL (range 0–100)**	**CAARS** ***t*-score** **(range 25–90)**	**WURS-k (range 0–84)**	**BDI-II (range 0–63)**	**BAI (range 0–63)**	**BSI,** **t-score (range 21–80)**	**DSM-IV, inattention (range 0–9)**	**DSM-IV, hyperactivity (range 0–9)**
1	21–25	3	2.43	108	–	–	10	37	**17**	22	33.3	**72**	5	**13**	9	**71**	2	0
1	21–25	5	3.19	184	–	–	3	10	8	29	32.4	60	22	**15**	**21**	**68**	**6**	2
1	21–25	8	4.66	<50.3	–	–	11	35	7	36	28.7	52	**32**	**13**	**10**	62	3	2
1	21–25	17	4.64	<50.3	+	–	9	21	**17**	29	28.7	63	25	**15**	**23**	**65**	4	4
1	26–30	20	4.18	<50.3	–	–	8	19	0	24	19.4	47	24	**12**	6	57	4	2
1	36–40	19	**7.36**	73.4	–	–	5	15	0	31	13	42	**31**	**17**	1	50	2	1
1	41–45	10	5.69	**201**	–	–	12	11	**12**	29	49	**75**	14	**29**	**12**	**80**	2	2
1	46–50	13	5.41	104	–	–	4	24	1	13	25	61	**40**	**16**	**31**	**80**	4	**8**
1	51–55	6	**8.57**	<50.3	–	–	10	28	12	24	13.9	46	**40**	9	**11**	60	**7**	5
1	51–55	16	**8.37**	74	–	–	7	18	11	26	13	n.a.^*^	**60**	**11**	**20**	**71**	**8**	**6**
**2**	21–25	2	4.43	**547**	–	Aripiprazole	6	15	1	20	31.5	n.a.^*^	7	**16**	0	49	2	1
**2**	26–30	7	2.50	**224**	–	–	7	18	0	34	19.4	**71**	**37**	8	**19**	61	2	**6**
**2**	41–45	14	6.07	66.6	–	–	5	19	**14**	20	10.3	28	**36**	4	4	57	**6**	4
**2**	61–65	12	2.76	71.6	–	–	6	18	1	22	28.7	58	14	**13**	**12**	**67**	0	0
4	<21	15	3.98	67	+	–	13	16	10	30	24.1	n.a.^*^	19	**27**	**17**	**80**	2	1
4	21–25	4	2.94	**455**	–	Dronabinol	5	25	9	29	22.2	61	18	0	3	56	5	2
4	36–40	9	5.13	141	–	Nabiximols	9	38	13	29	14.8	45	22	2	4	52	3	3
4	36–40	11	**6.65**	**387**	–	–	10	33	8	21	3.7	33	0	1	0	41	0	0
4	46–50	1	3.92	<50.3	–	–	5	26	5	19	6.5	57	14	0	8	56	4	5
4	56–60	18	4.74	<50.3	–	–	9	39	**15**	29	38	61	7	**15**	8	**66**	1	1

*M, male; f, female; OCB, oligoclonal bands; type 1, no OCB; type 2, positive OCB in CSF only; type 3, identical OCB in CSF and serum plus OCB in CSF only; type 4, identical OCB in CSF and serum*.

** *cut-off value: >200 IU/ml. Values in bold = exceeding cut-off values, ANA, antinuclear antibodies; YGTSS-TTS, Yale Global Tic Severity Scale—Total Tic Score; Y-BOCS, Yale Brown Obsessive Compulsive Scale; PUTS, Premonitory Urge for Tics Score; GTS-QoL, Gilles de la Tourette-Syndrome Quality of Life Scale (normalized); CAARS, Conner's Adult Attention deficit/hyperactivity disorder Rating Scale; n.a., not available*.

* *data not available due to high inconsistency index, WURS-k, Wender Utah Rating Scale short version; BDI-II, Beck Depression Inventory II; BAI, Beck Angst Inventory; BSI, Brief Symptom Inventory*.

### MRI

T1-weighted cerebral MRI did not reveal significant abnormalities suggesting increased intracranial pressure or an immunological or inflammatory disease in any of the patients.

### CSF Analysis

#### Routine Parameter

In none of the patients an elevated CSF cell count (≥5 cells/μl) was found. Using QAlb, CSF analyses showed a slightly dysfunctional blood-CSF-barrier in 4/20 patients (4/4 male patients, in none OCB type 2). In ten patients (50%), no OCB (type 1) could be detected. In four patients (20%, 2/4 male, 2/4 female), we found positive OCB in CSF only (type 2) indicating intrathecal IgG synthesis. One of these patients also showed an activated lympho-monocytic cell profile. None of the patients showed OCB type 3 (= combination of both identical OCB in CSF and serum and additionally OCB in CSF only). Identical OCB in serum and CSF (type 4) were observed in six patients (30%).

#### Detection of Specific CSF Autoantibodies

**Figure 1:**
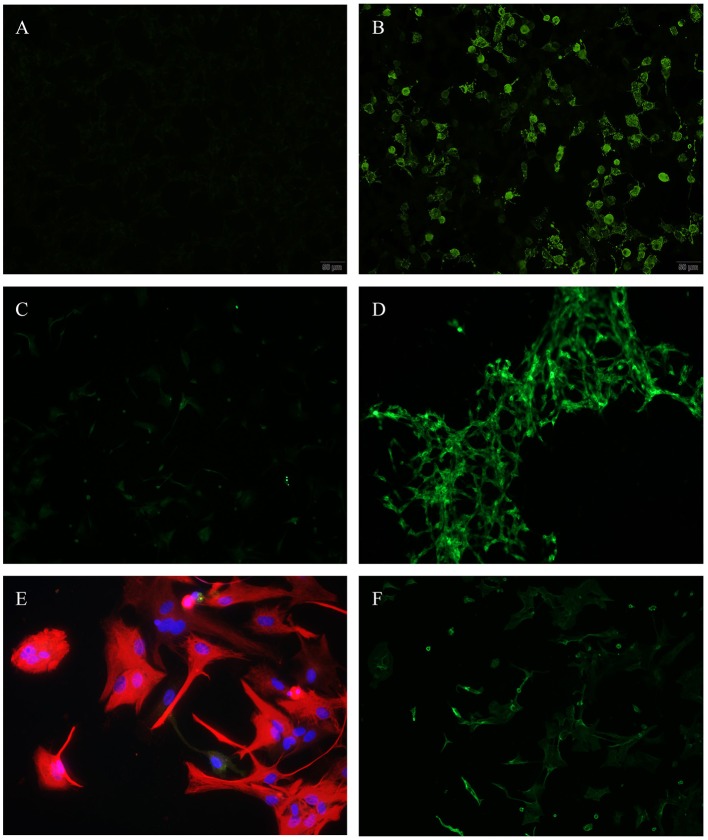
**(A,B)** Transfected HEK-cells, N-methyl-D-aspartate (NMDA), immunofluorescence, **(A)** incubated with patients' CSF—showing no antibody binding, **(B)** positive control. **(C,D)** Differentiated Glioma stem cells SY5Y (human), immunofluorescence, **(C)** incubated with patients' CSF—showing no antibody binding, **(D)** positive control. **(E,F)** Astrocytes (rat), **(E)** incubated with patients' CSF (red = anti glial fibrillary acidic protein (GFAP)—showing no antibody binding, **(F)** positive control (green = anti aquaporin 4).

Evaluation of the transfected HEK cells revealed no specific binding patterns to any of the expressed surface antigens as depicted in Figures 1A,B.

#### Detection of Unspecified CSF Autoantibodies

**Figure 2:**
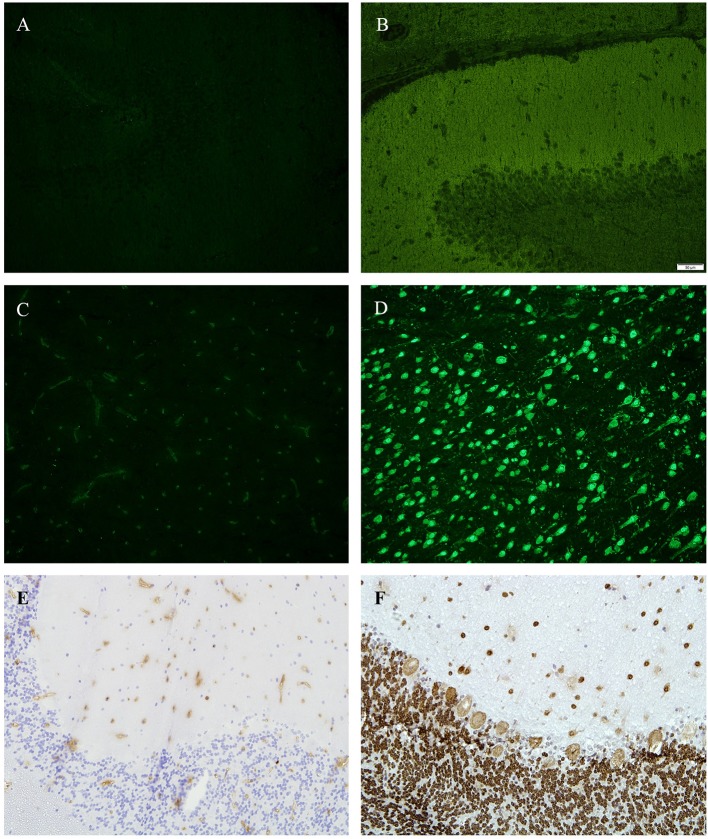
**(A,B)** Tissue sections from hippocampus (rat), immunofluorescence, **(A)** incubated with patients' CSF—showing no antibody binding, **(B)** positive control. **(C,D)** tissue sections from basal ganglia (rat), immunofluorescence, **(C)** incubated with patients' CSF—showing no antibody binding, **(D)** positive control (green = anti D2). **(E,F)** Tissue sections from cerebellum (monkey), 4′,6-diamidin-2-phenylindol (DAPI), **(A)** incubated with patients CSF—showing no antibody binding, **(E)** positive control (anti-Hu).

In two patients (10%), we identified positive ANA in sera and CSF (no OCB type 2), but failed to detect any other antibody binding pattern in any of the tissue sections (Figures 2A–D). By using enzyme linked immunohistochemistry on tissue-sections of monkey cerebellum, we were able to confirm results as seen in immunofluorescence (Figures 2E,F).

#### Glioma Stem Cell Line

To evaluate immune reactivity against different cells in the central nervous system, we used a human Glioma stem cell line (SY5Y). We detected positive ANA in the same two patients (10%), where we also identified positive ANA using immunoreactivity on tissue sections (see above). No other antibody binding was found as depicted in Figures 1C,D.

#### Glia Cells: Astrocytes

When comparing patients' CSF-incubated and dyed astrocytes to aquaporin 4 positive controls, we were unable to detect antibodies (Figures 1E,F). Presence of aquaporin 4 antibodies was additionally checked using transfected HEK cells providing the same negative results.

### Blood Sera

Measuring anti-streptolysin titers, five patients (25%) demonstrated elevated values (>200 IU/ml). Noteworthy, two of these patients (10%) also showed positive OCB type 2 in CSF and another two patients mirrored OCB in CSF and serum (pattern type 4). Only two patients (no. 9 and 13) reported a history of streptococcal infections in childhood. However, none of these showed elevated anti-streptolysin titers. None of the patient's sera demonstrated positive dopamine-2 antibodies.

### Relation of Clinical Data and CSF Findings

There were no statistically significant differences in mean age at tic onset between patients with positive OCB type 2 (age at tic onset: 6 years ± 0.81) and without OCB (age at tic onset: 8.1 years ±3 SD, *p* = 0.93). We also did not find any other significant differences between patients with and without positive OCB referring to tic severity (according to YGTSS-TTS), pre-monitory urges (PUTS), and comorbidities including OCD (Y-BOCS), ADHD (CAARS, WURS-K, DSM-IV symptom list), depression (BDI-II), anxiety (BAI) and psychological distress and psychiatric disorders (BSI) (for further details see Table 2).

**Table 2:** Relation between CSF findings and clinical data.

**Assessment**	**All patients (*N* = 20)**	**Patients without OCB (type 1 or 4) *N* = 16**	**Patients with positive OCB (type 2),** ***N* = 4**	**Mean difference**	***p*-value (patients with vs. patients without positive OCB)**
Age at lumbar puncture	36.1	35.4	38.7	+3.3	n.s.
Age at onset	7.7	8.1	6	−2.1	n.s.
YGTSS-TTS	23.2	24.7	17.5	−7.2	n.s.
Y-BOCS	8.0	9.1	4.0	−5.1	n.s.
PUTS	25.8	26.2	24.0	−2.2	n.s.
GTS-QOL	22.8	22.9	22.5	−0.4	n.s.
BDI-II	11.8	12.0	10.2	−1.8	n.s.
BAI	10.9	11.5	8.7	−2.7	n.s.
CAARS, t-score	54.8^*^	55.3^*^	52.3^*^	−3.0	n.s.
WURS-K	23.3	23.3	23.5	+0.2	n.s.
**DSM-IV symptom list**					
i. Inattention	3.3	3.6	2.5	−1.1	n.s.
ii. Hyperactivity	2.7	2.7	2.7	0.0	n.s.
BSI, t-score	62.5	63.3	58.5	−4.8	n.s

* *Results obtained from three patients could not be analyzed due to inconsistencies, n.s., not significant; YGTSS-TTS, Yale Global Tic Severity Scale—Total Tic Score; Y-BOCS, Yale Brown Obsessive Compulsive Scale; PUTS, Premonitory Urge for Tics Score; GTS-QoL, Gilles de la Tourette-Syndrome Quality of Life Scale (normalized); CAARS, Conner's Adult Attention deficit/hyperactivity disorder Rating Scale; WURS-k, Wender Utah Rating Scale short version; BDI-II, Beck Depression Inventory II; BAI, Beck Angst Inventory; BSI, Brief Symptom Inventory*.

## Discussion

The main result of this study was the finding of positive OCB type 2 in 4/20 of patients (20%) corroborating recent findings from our group in an independent sample of patients with GTS [positive OCB type 2 in 8/21 of patients (38%)] (24). However, the percentage of patients with positive OCB type 2 in this study was lower compared to our previous findings (20 vs. 38%). This could be explained either by the relatively small sample sizes or differences in clinical characteristics. For example, 16/21 patients in the study of Wenzel et al. (24)—but only 3/20 in this study—were medicated (e.g., antipsychotics, benzodiazepines, or serotonin reuptake inhibitors). Accordingly, one might speculate that patients included in this study suffered from less severe GTS. In addition, in this study, no children (age <18) were included, while Wenzel et al.'s sample comprised four children (9–17 years), of whom two showed positive OCB type 2. In contrast, in a recent study in five children with GTS none had positive OCB (17).

When combining results from our recent (24) and current studies (since samples were completely independent, but analyses were performed in the same lab), CSF OCB were positive in 29% (12/41 patients) of patients. Since in the general population positive OCB type 2 are found in only 5% of healthy people (25) and can be demonstrated only rarely in patients with non-inflammatory diseases (40, 41), our results clearly suggest a pathological immune process in terms of an intrathecal production of IgG antibodies. While in other diseases such as multiple sclerosis, an association between OCB and several inflammatory mediators linked to B-lymphocyte activity and other pro-inflammatory molecules has been demonstrated (42), in GTS such investigations have not been done yet. Since we detected OCB only in a subset of patients, from our results it is suggested that GTS represents a heterogeneous and multi-factorial disease. However, the clinical relevance of OCB in GTS needs further investigation as we were unable to find any differences between patients with and without positive OCB type 2 with respect to age, medication, age at tic onset, tic severity and number, kind and severity of comorbidities. Even a more detailed consideration of patients' history and clinical presentation did not reveal any conclusive clinical differences between these patients' groups. However, it should be kept in mind that in psychiatric diseases several different processes may trigger a B-cell response leading to the presence of CSF OCB (43).

Our findings are completely in line with results from a recent study based on the Swedish National Patient Register, where the authors describe a general increased risk (36%) for autoimmune diseases in patients with chronic tic disorders (44) and the data from the EMTICS study demonstrating altered levels of IL-6, TNF-α, TNF-receptor, and CD14 (21). Similar conclusions have been drawn in a review of 74 studies suggesting a connection between some autoimmune diseases and OCD/tic disorders (45). More specifically, previous data provided substantial evidence that in GTS a dysfunctional immune response is at least partially T-cell mediated [e.g., elevated TNF-α, IL-12 secretion (11)], while data supporting a B-cell driven, antibody mediated process is controversial, and a specific target antigen has not yet been identified (10). However, in all available studies autoantibody production has been assessed in patients' blood sera, but not CSF. Even though we were unable to detect a specific antibody in CSF, the high percentage of patients with CSF OCB suggests an involvement of a B-cell mediated immune response.

In two patients (10%) we detected positive ANA. Since the prevalence rate of positive ANA is about 27% in the general population (46) and because none of those patients exhibiting positive ANA also showed positive OCB or increased anti-streptolysin titers, this result can be considered as not pathological.

The second main result is the lack of autoantibodies detectable with the methods as described above. This is the first study investigating CSF samples for antibodies targeting both known and unknown antigens in a large number of adult patients with GTS. Although we used a wide spectrum of different methods including HEK cells expressing specific antigens, tissue sections of rat cerebellum, basal ganglia, and hippocampus as well as differentiated human Glioma stem cells and rat astrocytes, we failed to detect any CNS specific autoantibodies. In particular, we used not only tissue sections, but also living cells preventing possible loss of immunological reactivity to isolation or fixation processes. We primarily focused on IgG antibodies targeting cell surface antigens on live cells due to their functional impact on living cells.

This negative result can be interpreted in different ways: (i) a true absence of an autoantibody-related mechanism, at least directed to a single antigen; (ii) a failure to detect the target antigen on the cells and tissues used in this study, and (iii) the use of insufficient methods to detect autoantibodies.

Based on the autoimmune hypothesis for GTS, immunomodulatory interventions commonly used in autoimmune diseases have also been suggested for the treatment of GTS including plasmapheresis (47–49), the non-steroidal anti-inflammatory drug celecoxib (50), and intravenous immunoglobulin (IVIG) (47, 49, 51–54). Available studies resulted in conflicting findings with positive (47–50, 52–54) and negative results (51). However, all these studies are limited by the fact that no pre-selection of patients was performed depending on a marker indicating increased immune activation. It can be hypothesized that immunomodulatory treatments might be effective only in a subgroup of patients with underlying (auto)immunity (55, 56). We suggest that positive OCB may serve as such a marker to identify patients with an immune activation who might benefit from immunomodulatory treatments.

The following limitations of our study have to be addressed: (i) the sample size was quite small. However, relatively high effort and the invasive procedure related to lumbar puncture have to be taken into account as well as the fact that in clinical routine CSF analysis is not recommended in patients with tic disorders. When combining data from our two independent samples in GTS, our sample consists of 41 patients, of whom nearly one third exhibits positive OCB; (ii) although patients received a compensation fee for participating in the study, we do not believe that this has caused any bias in patient selection; and (iii) antibody binding was not tested on human brain sections. Yet this is due to the limited availability of these sections. A possible approach to margin this limitation is the use of CSF on protein arrays containing large numbers of CNS proteins. Furthermore, the prior fixation of tissue might have biased the results by possible loss of immunoreactivity. Data presented are based on immunochemistry results. Antibody detection via fluorescent cell sorting is possible (23) yet the clinical significance of low-level antibodies possibly detected by this is unclear. Therefore, for routine diagnostic of CNS autoantibodies mainly immunofluorescence or blot techniques are used.

In conclusion, although we failed to detect any specific autoantibodies, our finding of positive CSF OCB in a subset of patients points a possible humoral immune contribution to GTS and therefore supports the assumption of autoimmune processes being involved.

## Data Availability

All datasets for this study are included in the manuscript and the supplementary files.

## Ethics Statement

This study was carried out in accordance with the recommendations of the Local Ethics Committee (no. 6987) of the Hannover Medical School with written informed consent from all subjects. All subjects gave written informed consent in accordance with the Declaration of Helsinki. The protocol was approved by the Local Ethics Committee of the Hannover Medical School.

## Author Contributions

KM-V and K-WS contributed conception and design of the study. CB, KM-V, K-WS, JK, PS, TS, MS, and CS contributed acquisition of data and organized the database. CB wrote the first draft of the manuscript. All authors contributed analysis and interpretation of data and contributed to manuscript revision, read, and approved the submitted version.

### Conflict of Interest Statement

The authors declare that the research was conducted in the absence of any commercial or financial relationships that could be construed as a potential conflict of interest.
